# ﻿*Paucibranchiaglemareci* sp. nov. (Annelida, Eunicidae), a new species from the French Atlantic continental shelf

**DOI:** 10.3897/zookeys.1232.143944

**Published:** 2025-03-18

**Authors:** Lucas Pinsivy, Nicolas Lavesque, Guillemine Daffe, Flore Daramy, Pat Hutchings

**Affiliations:** 1 UAR 3113, Observatoire Marin, Université de Brest, 29280 Plouzané, France Université de Brest Plouzané France; 2 Laboratoire des Sciences de l’Environnement Marin (LEMAR), UMR6539 CNRS/UBO/IRD/IFREMER, Plouzané, France Laboratoire des Sciences de l’Environnement Marin Plouzané France; 3 Université de Bordeaux, CNRS, Bordeaux INP, EPOC, UMR 5805, Arcachon, France Université de Bordeaux Arcachon France; 4 CNRS, Université de Bordeaux – Observatoire Aquitain des Sciences de l’Univers, UAR 2567, POREA, Pessac, France Université de Bordeaux Pessac France; 5 Australian Museum Research Institute, Australian Museum, NSW 2010, Sydney, Australia Australian Museum Sydney Australia; 6 Marine Ecology Group, School of Natural Sciences, Faculty of Science and Engineering, Wallumattagal Campus, Macquarie University, NSW 2109, Australia Macquarie University Sydney Australia

**Keywords:** Bay of Biscay, COI, morphology, taxonomic key

## Abstract

In this study, we describe a new species belonging to the genus *Paucibranchia* Molina-Acevedo, 2018 found in the area “La Grande Vasière” on the French Atlantic continental shelf (Bay of Biscay). *Paucibranchiaglemareci***sp. nov.** lives between 100 and 130 m depth on muddy sands. It is easily distinguished from most other European species of the genus by the absence of compound spinigerous chaetae. A key to the European species of the genus *Paucibranchia* is given.

## ﻿Introduction

The genus *Paucibranchia* was erected to include species of the genus *Marphysa* Quatrefages, 1865, which is characterized by branchiae restricted to a short anterior region of the body (Molina-Acevedo 2018). This group of species was already recognized as a subgroup of *Marphysa* by Fauchald in his study of the order Eunicida (Fauchald 1970) and by Orensanz in his study of the Antarctic and Subantarctic “Eunicemorph” polychaetes (Orensanz 1990).

Worldwide, the genus *Paucibranchia* includes 19 accepted species (Read and Fauchald 2025), with the type species, *Paucibranchiabellii* (Audouin & Milne Edwards, 1833), described from the Chausey Islands in the English Channel. Among them, eight species possess only compound falcigers, whereas all other species have compound spinigers or both falcigers and spinigers.

Several species have been reported from European waters including *P.adenensis* (Gravier, 1900) (type locality: Gulf of Aden, Yemen, Indian Ocean), *P.bellii* (type locality: Chausey Islands, France, English Channel), *P.cinari* (Kurt-Sahin, 2014) (type locality: Sea of Marmara, Turkey, Mediterranean Sea), *P.fallax* (Marion & Bobretzky, 1875) (type locality: Gulf of Marseille, France, Mediterranean Sea), *P.kinbergi* (McIntosh, 1910) (type locality: off Cap Finisterre, Spain, Atlantic Ocean), and *P.totospinata* (Lu & Fauchald, 1998) (type locality: near Blacksod Bay, Ireland, Atlantic Ocean). *Paucibranchiaadenensis* is the only species characterised by having only compound falcigers in European waters. This species, was originally described from the Gulf of Aden and has since been recorded (questionably) within the Mediterranean Sea, from Levantine Sea to the Tyrrhenian Sea (Katsiaras et al. 2014; Katsiaras 2021; Rousou et al. 2023; Langeneck et al. 2024).

In this study, we describe a second European species of the genus *Paucibranchia* having only compound falcigers. It is the first species known from the North-East Atlantic.

## ﻿Materials and methods

### ﻿Sampling and morphological analyses

**Figure 1.**
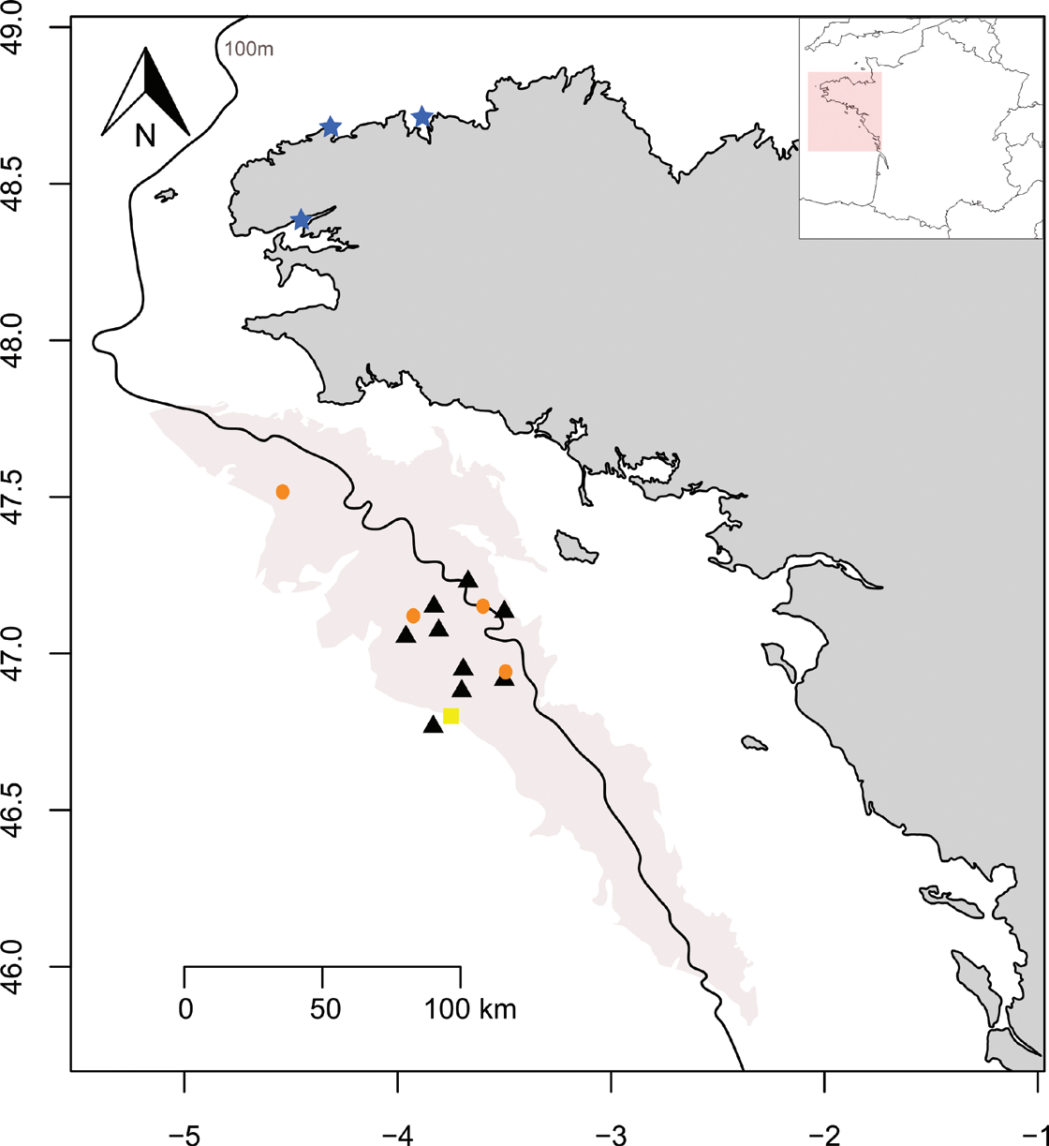
Sampling localities of *Paucibranchiaglemareci* sp. nov. on the area of the “Grande Vasière” (in grey on the map). Yellow squares: holotype, orange circles: paratypes, black triangles: additional material collected. The blue stars indicate the sampling localities of the *Paucibranchiabellii* specimens analysed in this study.

Specimens of the new species where collected from the northern Bay of Biscay (Fig. 1) during the oceanographic cruises APPEAL ATL 19-1 (https://doi.org/10.17600/18001040), APPEAL ATL 19-2 (https://doi.org/10.17600/18001062), and EVHOE 2022 (https://doi.org/10.17600/18001822) in May 2019, September 2019 and October 2022, respectively, using grabs and Rallier du Baty dredges. Samples were fixed in 4% formaldehyde–seawater solution and later transferred to 70% ethanol or directly sieved and frozen onboard for molecular analyses. Specimens of *Paucibranchiabellii* matching well the redescription of this species by Molina-Acevedo (2018) were collected on Brittany’s shore for molecular analyses (Table 1, Fig. 1). These specimens were sampled using grabs or hand corer in 2022 or 2023 and fixed in 70% ethanol.

**Table 1.** Terminal taxa used in molecular part of the study (COI gene), with type localities, collection localities, GenBank accession numbers and references.

Species	Type locality	Collection locality	GenBank accession no.	Reference
Eunicecf.violaceomaculata	Tortugas, Caribbean	Carrie Bow Cay, Belize	GQ497542	Zanol et al. 2010
*Palolaviridis*	Samoa, Pacific Ocean	Kosrae, Micronesia	GQ497556	Zanol et al. 2010
*Leodicerubra*	Saint Thomas, Caribbean	Ceara, Brazil	GQ497528	Zanol et al. 2010
*M.aegypti*	Suez Canal, Egypt	Suez Canal, Egypt	MF196969	Elgetany et al. 2018
*M.bifurcata*	WA, Australia	Qld, Australia	KX172177	Zanol et al. 2016
*M.bifurcata*	WA, Australia	Qld, Australia	KX172178	Zanol et al. 2016
*M.brevitentaculata*	Tobago	Quintana Roo, Mexico	GQ497548	Zanol et al. 2010
*M.californica*	California, USA	California, USA	GQ497552	Zanol et al. 2010
*M.chirigota*	Bay of Cadiz, Spain	Bay of Cadiz, Spain	MN816442	Martin et al. 2020
*M.chirigota*	Bay of Cadiz, Spain	Bay of Cadiz, Spain	MN816443	Martin et al. 2020
*M.chirigota*	Bay of Cadiz, Spain	Bay of Cadiz, Spain	MN816444	Martin et al. 2020
*M.davidattenboroughi*	Bass Strait, Australia	Bass Strait, Australia	OQ622195	Lavesque et al. 2023
*M.davidattenboroughi*	Bass Strait, Australia	Bass Strait, Australia	OQ622196	Lavesque et al. 2023
*M.davidattenboroughi*	Bass Strait, Australia	Bass Strait, Australia	OQ622197	Lavesque et al. 2023
*M.davidattenboroughi*	Bass Strait, Australia	Bass Strait, Australia	OQ622198	Lavesque et al. 2023
*M.davidattenboroughi*	Bass Strait, Australia	Bass Strait, Australia	OQ622199	Lavesque et al. 2023
*M.fauchaldi*	NT, Australia	NT, Australia	KX172165	Zanol et al. 2016
*M.gaditana*	Bay of Cadiz, Spain	Bay of Cadiz, Spain	MN816441	Martin et al. 2020
*M.hongkongensa*	Hong Kong	Hong Kong	MH598525	Wang et al. 2018
*M.hongkongensa*	Hong Kong	Hong Kong	MH598526	Wang et al. 2018
*M.iloiloensis*	Iloilo, Philippines	Tigbauan, Philippines	MN106279	Glasby et al. 2019
*M.iloiloensis*	Iloilo, Philippines	Tigbauan, Philippines	MN106280	Glasby et al. 2019
*M.iloiloensis*	Iloilo, Philippines	Tigbauan, Philippines	MN106281	Glasby et al. 2019
*M.kristiani*	NSW, Australia	NSW, Australia	KX172160	Zanol et al. 2016
*M.kristiani*	NSW, Australia	NSW, Australia	KX172161	Zanol et al. 2016
*M.kristiani*	NSW, Australia	NSW, Australia	KX172162	Zanol et al. 2016
*M.kristiani*	NSW, Australia	NSW, Australia	KX172158	Zanol et al. 2016
*M.madrasi*	Chennai, India	Chennai, India	MT813506	Hutchings et al. 2020
*M.madrasi*	Chennai, India	Chennai, India	MT813507	Hutchings et al. 2020
*M.mossambica*	Mozambique	Iloilo, Philippines	KX172164	Zanol et al. 2016
*M.mullawa*	Qld, Australia	NSW, Australia	KX172166	Zanol et al. 2016
*M.mullawa*	Qld, Australia	NSW, Australia	KX172167	Zanol et al. 2016
*M.mullawa*	Qld, Australia	NSW, Australia	KX172168	Zanol et al. 2016
*M.mullawa*	Qld, Australia	NSW, Australia	KX172176	Zanol et al. 2016
*M.papuaensis*	Papua New Guinea	Papua New Guinea	OP184050	Lavesque et al. 2022
*M.pseudosessiloa*	NSW, Australia	NSW, Australia	KY605405	Zanol et al. 2010
*M.pseudosessiloa*	NSW, Australia	NSW, Australia	KY605406	Zanol et al. 2010
*M.regalis*	Bermuda	Ceara, Brazil	GQ497562	Zanol et al. 2010
*M.sanguinea*	Devon, UK	Callot Island, France	GQ497547	Zanol et al. 2010
*M.sanguinea*	Devon, UK	Cornwall, UK	MK541904	Lavesque et al. 2019
*M.sanguinea*	Devon, UK	Arcachon Bay, France	MK950853	Lavesque et al. 2019
*M.sanguinea*	Devon, UK	Brest, France	MK967470	Lavesque et al. 2019
*M.sherlockae*	Durban, South Africa	Strand, South Africa	MT840349	Kara et al. 2020
*M.sherlockae*	Durban, South Africa	Strand, South Africa	MT840350	Kara et al. 2020
*M.sherlockae*	Durban, South Africa	Strand, South Africa	MT840351	Kara et al. 2020
*M.tripectinata*	Beihai, China	Beihai, China	MN106271	Glasby et al. 2019
*M.tripectinata*	Beihai, China	Beihai, China	MN106272	Glasby et al. 2019
*M.tripectinata*	Beihai, China	Beihai, China	MN106273	Glasby et al. 2019
*M.tripectinata*	Beihai, China	Beihai, China	MN106274	Glasby et al. 2019
*M.tripectinata*	Beihai, China	Beihai, China	MN106275	Glasby et al. 2019
*M.tripectinata*	Beihai, China	Beihai, China	MN106276	Glasby et al. 2019
*M.tripectinata*	Beihai, China	Beihai, China	MN106277	Glasby et al. 2019
*M.tripectinata*	Beihai, China	Beihai, China	MN106278	Glasby et al. 2019
*M.victori*	Arcachon Bay, France	Arcachon Bay, France	MG384996	Lavesque et al. 2017
*M.victori*	Arcachon Bay, France	Arcachon Bay, France	MG384997	Lavesque et al. 2017
*M.victori*	Arcachon Bay, France	Arcachon Bay, France	MG384998	Lavesque et al. 2017
*M.victori*	Arcachon Bay, France	Arcachon Bay, France	MG384999	Lavesque et al. 2017
*M.victori*	Arcachon Bay, France	Mangoku-ura Inlet, Japan	LC467767	Abe et al. 2019
*M.victori*	Arcachon Bay, France	Sendai Bay, Japan	LC467769	Abe et al. 2019
*M.victori*	Arcachon Bay, France	Ena Bay, Japan	LC467772	Abe et al. 2019
*M.victori*	Arcachon Bay, France	China	MT012514	Lavesque et al. 2020
*M.viridis*	Florida, USA	Ceara, Brazil	GQ497553	Zanol et al. 2010
*M.zanolae*	Papua New Guinea	Papua New Guinea	OP184049	Lavesque et al. 2022
*P.bellii*	France	France, Brittany	PV019092	This study
*P.bellii*	France	France, Brittany	PV019093	This study
*P.bellii*	France	France, Brittany	PV019094	This study
*P.bellii*	France	France, Brittany	PV019095	This study
*P.disjuncta*	California, USA	California, USA	GQ497549	Zanol et al. 2010
*P.glemareci* sp. nov.	France, Brittany	France, Brittany	PV021094	This study
*P.triantennata*	Korean Peninsula	Korean Peninsula	OM158712	Kim et al. 2022
*P.triantennata*	Korean Peninsula	Korean Peninsula	OM158713	Kim et al. 2022

Preserved specimens were examined under a Nikon SMZ25 stereomicroscope and a Nikon Eclipse E400 microscope and photographed with a Nikon DS-Ri 2 camera. Measurements were made with the NIS-Elements Analysis software. Map was made using R v. 4.4.1 statistical software (R Core Team 2024) and the “maps” v. 3.4.2 package (Becker et al. 2023). Information on bathymetry was provided by EMODnet Digital Bathymetry (DTM 2022). Drawings of parapodia were made using Inkscape software. For SEM pictures, selected parapodia along the body were removed from a paratype (AM W.55323), dehydrated in ethanol, critical-point dried, covered with 20 nm of gold, examined under the scanning electron microscope (JEOL JSM 6480LA) and imaged with a secondary detector at Macquarie University, Sydney, Australia.

The terminology used to describe jaws morphology follows Molina-Acevedo and Carrera-Parra (2015) and the terminology of pectinate chaetae follows Carrera-Parra and Salazar-Vallejo (1998) for the relative length of external and internal teeth, Zanol et al. (2014, 2016) for the thickness of the shaft, Molina-Acevedo and Carrera-Parra (2015) for the thickness of the blade, and Glasby et al. (2019) for the size of internal teeth.

### ﻿Repositories

The studied material is deposited at the Australian Museum, Sydney, Australia (**AM**), the Muséum national d’Histoire naturelle, Paris, France (**MNHN**), and the Station Marine d’Arcachon, Arcachon, France (**SMA**).

### ﻿Molecular analyses

Extraction of DNA was done with Maxwell (Promega), an automated DNA/RNA isolation, with Maxwell® RSC Blood DNA kit, following protocol supplied by the manufacturers. Approximately 600 bp of the COI (cytochrome c oxidase subunit I) gene were amplified, using the primers polyLCO and polyHCO (Carr et al. 2011). Polymerase Chain Reaction (PCR) was performed with GoTaq® G2 Flexi DNA Polymerase Kit in 20 μL mixtures containing: 4 μL of 5× Green GoTaq® Flexi Reaction Buffer (final concentration of 1×), 1.2 μL of MgCl2 (25 Mm) solution, 0.4 μL of PCR nucleotide mix (final concentration of 0.2 mM each dNTP), 0.2 μl of each primer (final concentration of 1 μM), 0.1 μl of Taq DNA Polymerase (5U/μl), 1 μl template DNA and 12.9 μL of nuclease-free water. The temperature profile was as follows 94 °C / 300 s – (94 °C / 40 s–45 °C / 40 s–72 °C / 1 min)*5 cycles – (94 °C / 40 s–51 °C/ 40 s–72 °C / 1 min)*35 cycles – 72 °C / 300 s – 4 °C. Amplified products were sent to Eurofins Genomics Company to complete double strain sequencing, using same set of primers as used for PCR. Forward and reverse sequence fragments were merged into consensus sequences.

A total of 65 COI sequences were downloaded from GenBank and five sequences obtained during this study, with 67 sequences belonging to *Marphysa* or *Paucibranchia* species, whilst the remaining three were of closely related genera within the Eunicidae and used as outgroups (Table 1). All COI sequences were aligned in Geneious Prime 2025.0.2 using the MUSCLE plugin and default settings. The maximum-likelihood analysis was performed in IQ-TREE 2.2.0 (Trifinopoulos et al. 2016) with the best fitting evolutionary model TIM2+F+I+G4 selected. Bootstrap support was estimated using an ultrafast bootstrap algorithm (UFBoot) (Minh et al. 2013) for 1000 replicates. Pair-wise Kimura 2-parameter (K2P) genetic distance was performed using MEGA v. 7.0.26.

### ﻿Taxonomic account

**Family Eunicidae Berthold, 1827**

#### ﻿Genus Paucibranchia

Taxon classificationAnimaliaEunicidaEunicidae

Molina-Acevedo, 2018

D938C3BA-166C-52DE-AE17-462C5AC32F7D

##### Type species.

*Paucibranchiabellii* (Audouin & Milne Edwards, 1833) (type by original designation).

#### ﻿ Paucibranchia glemareci sp. nov.

Taxon classificationAnimaliaEunicidaEunicidae

4EAC680D-208B-56DA-8ED5-AA1DC8F71899

https://zoobank.org/5D00BBF5-7D2A-4821-A913-92864E2394ED

Figs 2 , 3 , 4

##### Material examined.

***Holotype*.** France – **Bay of Biscay** • “Grande Vasière”; 46.800, −3.750; depth 128 m; Sep. 2019; APPEAL ATL 19-2 Campaign; station FLLD2; Hamon grab; MNHN-IA 2000-2112 ***Paratypes*.** France – **Bay of Biscay** • 3 specimens; same collection data as for the holotype; MNHN-IA 2000-2113 and MNHN-IA 2000-2114, AM W.55320 • 1 spec.; “Grande Vasière”; 47.116, −3.910; depth 116 m; Sep. 2019; APPEAL ATL 19-2 Campaign; stn FLLC3; Hamon grab; MNHN-IA 2000-2115 • 1 spec.; “Grande Vasière”; 46.940, −3.480; depth 109 m; Sep. 2019; APPEAL ATL 19-2 Campaign; stn FLLBB1; Hamon grab; MNHN-IA 2000-2116 • 1 spec.; “Grande Vasière”; 47.150, −3.587; depth 101 m; May 2019; APPEAL ATL 19-1 Campaign; stn 5880; Rallier du Baty dredge; MNHN-IA 2000-2117 • 1 spec. (mounted for SEM); same data as for MNHN-IA 2000-2117; AM W.55323.

##### Additional material for molecular analyses.

*Paucibranchiaglemareci* sp. nov. France – **Bay of Biscay** • 1 spec.; “Grande Vasière”; 47.514, −4.540; depth 112 m; Oct. 2022; EVHOE 2022 Campaign; stn A1470; Rallier du Baty dredge; GenBank no: PV021094 (COI); SMA-NL298 *Paucibranchiabellii*. France – **Brittany** • 1 spec.; Morlaix Bay, Pierre Noire; 48.708, −3.866; depth 17 m; Feb. 2023; Céline Houbin leg.; Van Veen grab; GenBank no: PV019095 (COI); SMA-NL194 • 2 specs; Brignogan; 48.673, −4.321; Intertidal; Mar. 2023; Jacques Grall leg.; Hand corer; GenBank no: PV019094, PV019092 (COI); SMA-NL192 and SMA-NL183 • 1 spec.; Brest, “du Château” harbour; 48.378, −4.488; depth 5 m; Jul. 2022; Vincent Le Garrec leg.; Day grab; GenBank no: PV019093 (COI); SMA-NL185.

##### Diagnosis.

Prostomium anteriorly rounded with ventral sulcus. Five prostomial appendages arranged in an arc on posterior margin of prostomium. Eyes present, round and dark. Maxillary formula: 1+1, 8+9(8), 9+0, 5(6)+10, 1+1. Branchiae pectinate, present from chaetiger 14–16 to 31–33 with 8–13 long filaments. Dorsal cirri always well developed, slightly longer but thinner in postbranchial chaetigers. Ventral cirri shorter than dorsal cirri, bluntly triangular in pre-branchial region, becoming bluntly conical and thinner through the body. Postchaetal lobes well developed in anterior part of body, becoming inconspicuous from about chaetiger 45 onwards. Three or four light brown aciculae in prebranchial chaetigers, decreasing to two in anterior part of branchial region and to one thereafter. Subacicular hooks light brown, bidentate, commencing from chaetiger 33–37 and present in all following chaetigers; most often one per chaetiger but sometimes two in posterior part of body. Compound chaetae all bidentate falcigers, with two sizes of blades, short ones about 50 µm, long ones about 90 µm. One type of pectinate chaetae identified: narrow, isodont with 2–5 long and slender internal teeth. Posterior pectinate chaetae, if different, unknown. Pygidium unknown.

##### Description

**Figure 2.**
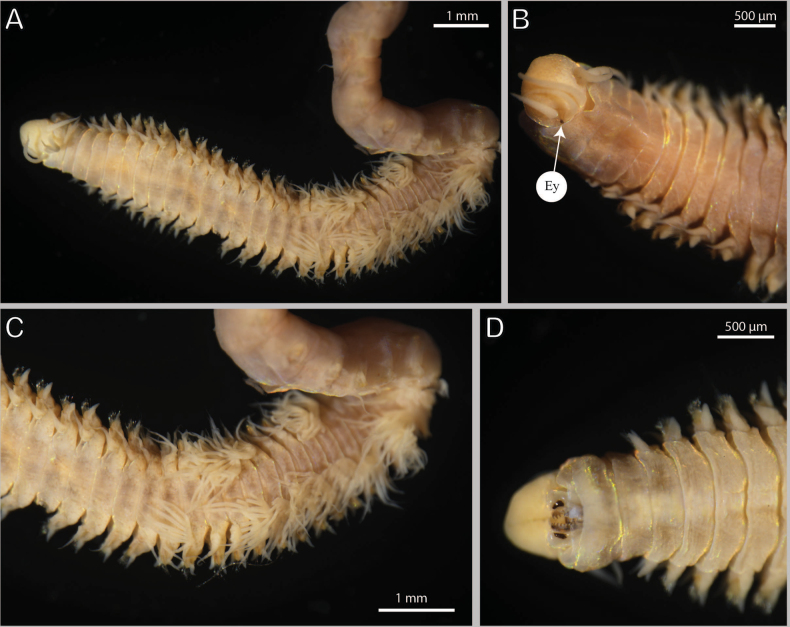
*Paucibranchiaglemareci* sp. nov. paratype MNHN-IA 2000-2113 (**A, C, D**), holotype MNHN-IA 2000-2112 (**B**) **A** anterior end, dorsal view **B** anterior end, dorsal view **C** branchial chaetigers, dorsal view **D** anterior end, ventral view. Abbreviations: Ey, eyes.

(based on holotype, with variation in parentheses for paratypes). Specimens fixed in alcohol whitish, specimens fixed in formalin pinkish with reddish spots on prostomium, ventrum, parapodia, dorsal cirri, and lateral parts of dorsum (Fig. 2B, C). All specimens incomplete, holotype with 60 chaetigers (longest paratype with about 90 chaetigers), about 26 mm (13.3–47.5 mm) long, 1.9 mm (1.0–2.5 mm) wide at chaetiger 10, including parapodia. Body round in cross section anteriorly (about chaetiger 7–8), dorsoventrally flattened thereafter.

Prostomium anteriorly rounded (slightly conical), without dorsal median sulcus, ventral sulcus deep (Figs 2A–D, 4A, B). Palps and antennae arranged in an arc on posterior margin of prostomium. Median antenna isolated by gap from lateral antennae and palps. Median antenna longer than lateral ones, lateral antennae longer than palps, antennae much longer and palps slightly longer (same size) than prostomium (Figs 2B, 4A). Median antenna reaching chaetiger 3 (2), lateral antennae end of chaetiger 1 (end of second peristomial ring) and palps second peristomial ring (end of first peristomial ring) (Figs 2B, 4A). Ceratostyles and palpostyles slender and tapering, with indistinct cylindrical articulations. Ceratophores and palpophores indistinct. Eyes present, one pair, rounded, black, situated at posterior base of palps and lateral to lateral antennae (Fig. 2B). Separation between both peristomial rings distinct on all sides. First peristomial ring as long as second one dorsally (1½ × as long as second one), twice as long laterally (Figs 2B, 4A). Anterior dorsal margin of first peristomial ring forming convoluting collar on holotype and most paratypes (Fig. 2B). Some small specimens (non-type), less than 1 mm wide, lacking palps.

**Figure 3.**
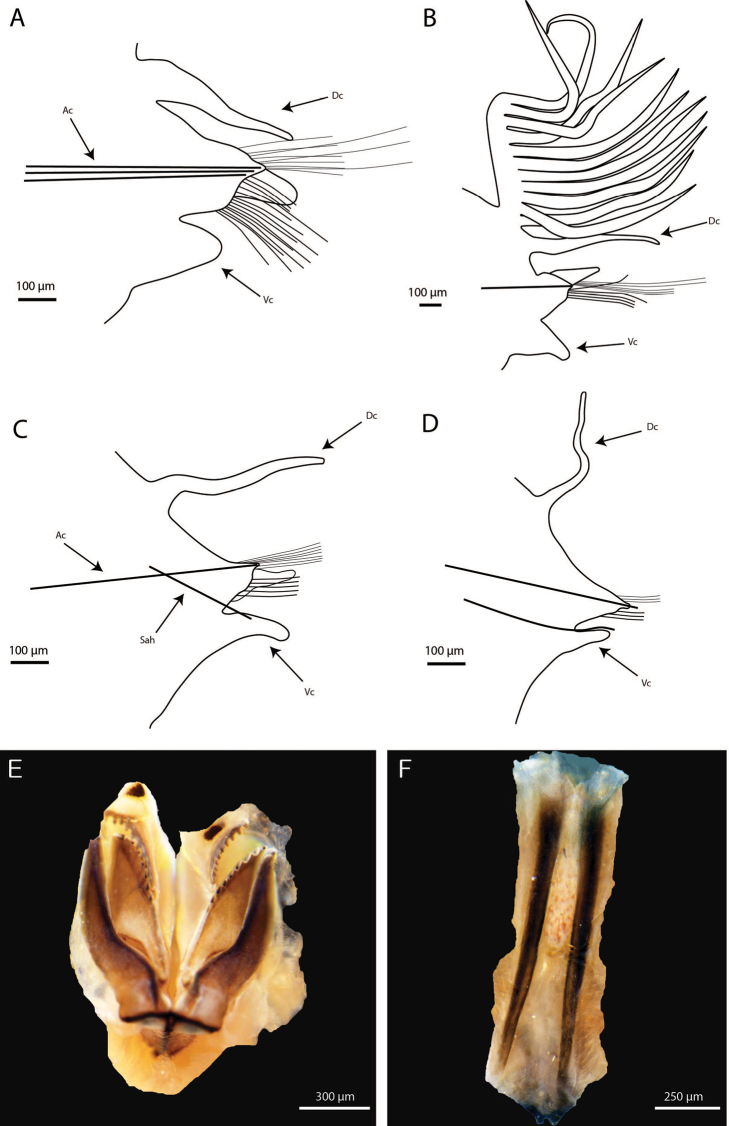
*Paucibranchiaglemareci* sp. nov. paratype MNHN-IA 2000-2116 (**A, D**), paratype MNHN-IA 2000-2114 (**B, C, E, F**) **A** parapodia from chaetiger 10 **B** parapodia from chaetiger 22 **C** parapodia from chaetiger 40 **D** parapodia from chaetiger 87 **E** maxilla, dorsal view **F** mandibles, dorsal view. Abbreviations: Ac, acicula; Dc, dorsal cirri; Sah, sub-acicular hook; Vc, ventral cirri.

Maxillary formula as follows: MF = 1+1, 8+9(8), 9+0, 5(6)+10, 1+1, MVI absent (Fig. 3E). Maxillary carrier approximately 2× shorter than MI, rectangular anteriorly, triangular posteriorly, with a pair of rounded wings situated at posterolateral margins. MI forceps-like, without attachment lamellae, with falcal arch developed, rounded; with outer edge of base straight and with curvature in basal inner edge where base of maxillae II is supported. Closing system approximately 5× shorter than MI. MII without attachment lamella but with small basal ligament, teeth triangular, distributed on half of plate length. MIII, single, longer than left MIV, slightly curved, with equal-sized triangular teeth, without attachment lamella but with small basal ligament. Left MIV short (less than half length of right MIV), attachment lamella dark, 2× shorter than corresponding MIV, subtriangular. Right MIV long, with teeth triangular, decreasing in size posteriorly; attachment lamella oval, 3× shorter than corresponding MIV, dark. MV, paired, longer than high, whitish (Fig. 3E). Mandibles light brown, with concentric stripes; longer than MI; cutting plates whitish (Fig. 3F).

**Figure 4.**
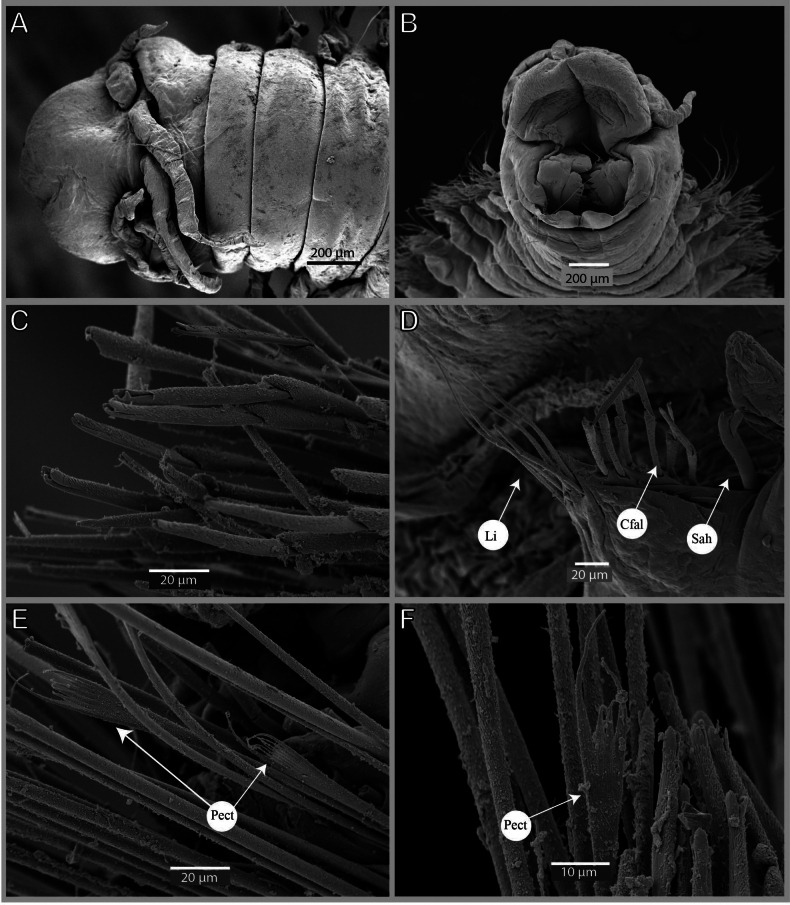
*Paucibranchiaglemareci* sp. nov. paratype AM W.55323, SEM **A** anterior end, dorsal view **B** anterior end, ventral view **C** compound falcigers, chaetiger 9 **D** parapodia, chaetiger 47 **E** pectinate chaetae, chaetiger 6 **F** pectinate chaetae, chaetiger 2. Abbreviations: Cfal, compound falciger; Li, limbate chaetae; Pect, pectinate chaetae; Sah, Sub-acicular hook.

First three parapodia smallest; most developed from chaetiger 4 to end of branchial chaetigers, following ones becoming gradually smaller (Fig. 3A–D). Prechaetal lobes as transverse fold in all chaetigers. Postchaetal lobes well developed until end of branchial chaetigers, bluntly triangular in first 9–10 chaetigers, becoming conical, longer and thinner through branchial region, then decreasing in size, becoming inconspicuous from about chaetiger 45. Dorsal cirri conical, tapering, becoming slender and longer from first chaetiger to end of branchial region, then filiform until end of body. Dorsal cirri slightly longer in post-branchial region than in pre-branchial chaetigers. Ventral cirri shorter than dorsal cirri, bluntly triangular in pre-branchial region, becoming bluntly conical and thinner throughout body (Fig. 3A–D).

Branchiae pectinate, commencing from chaetiger 16 (14–15) continuing for a limited number of segments, until chaetiger 32 (31–33); with 8–13 long filaments; branchial filament about 1.5× longer than dorsal cirri where best developed (Figs 2C, 3B). Smaller specimens (non-type) have branchiae starting earlier and less numerous (from chaetiger 11 to 20 for a specimen 0.8 mm wide).

Aciculae light brown with paler blunt tips, three or four aciculae on pre-branchial chaetigers, two on anterior part of branchial region, and one from mid part of branchial region and following chaetigers; some posterior chaetigers with two aciculae. Supra-acicular chaetae with limbate capillaries and pectinates; capillaries present from first chaetiger to posterior ones, numbering up to 15 in anterior chaetigers and up to five in posteriormost chaetigers (Fig. 4C, D). One type of pectinate chaetae identified: narrow, isodont with 2–5 long and slender internal teeth; inner teeth with terminal filaments; outer teeth longer, but of different length (Fig. 4E, F), anterior body with two or three pectinate chaetae by parapodium, mid-body chaetigers with one chaeta, not seen posteriorly (but longest paratype with most of posterior chaetae broken). Subacicular chaetae including compound falcigers and subacicular hooks, compound spinigers absent (Fig. 4C, D). Compound falcigers bidentate, with two sizes of blades, short ones about 50 µm, long ones about 90 µm, commencing from first chaetiger to posterior part, with more than 30 chaetae within parapodium in anterior part, with about seven chaetae in mid-body and four or five on last chaetigers (Fig. 4C, D). Subacicular hooks (SAH) light brown, bidentate, commencing from chaetiger 35 (33–37) and present in all chaetigers thereafter, ventral to bundle of falcigers, generally one per parapodium; few posterior chaetigers with two hooks (Fig. 4D). Smaller specimens (non-type) have SAH starting earlier (at 19^th^ chaetiger for a specimen of 0.6 mm wide and at 29^th^ for a specimen 0.8 mm wide).

Pygidium unknown.

##### Etymology.

This species is named after Michel Glemarec for his major contribution to the ecology of the Grande Vasière and the taxonomy of polychaetes.

##### Type locality.

Northeastern Atlantic Ocean, Bay of Biscay, “Grande Vasière”, Station FLLD2 (46.800, −3.750, 128 m depth).

##### Distribution.

Known from the “Grande Vasière” area.

##### Habitat.

Fine sands to muddy sands, between 100 and 130 m depth.

##### Remarks.

*Paucibranchiaglemareci* sp. nov. is easily distinguished from other species described from Europe by the presence of compound falcigers and the absence of compound spinigers. It is, however, very close to *P.adenensis* (Gravier, 1900), described from Yemen and (questionably) later reported from the Mediterranean Sea (Katsiaras et al. 2014; Katsiaras 2021; Rousou et al. 2023; Langeneck et al. 2024). These two species share the presence of rounded eyes, only falcigerous compound chaetae, same branchial distribution and similar looking bidentate sub-acicular hooks. They can however be separated based on morphological characters. According to the redescription of *P.adenensis* by Molina-Acevedo (2018), *P.glemareci* sp. nov. differs by its maxillary formula (1+1, 8+9(8), 9+0, 5(6)+10, 1+1) versus (1+1, 7(8)+7(8), 6(7)+0, 4(5)+7(8-9), 1+1) for *P.adenensis*, the number of aciculae on prebranchial chaetigers (up to four for *P.glemareci* sp. nov. versus up to two for *P.adenensis*) and shorter blades on anterior compound falcigers (50 and 90 µm for *P.glemareci* sp. nov. versus 90 and 105 µm for *P.adenensis*). Moreover, both species appear to have a different colouration after fixation; *P.glemareci* sp. nov. presents reddish spots on most specimens, but never brown colouration as observed on some non-type specimens of *P.adenensis* by Molina-Acevedo (2018).

Worldwide, *P.glemareci* sp. nov. shares the absence of compound spinigers with *P.conferta* (Moore, 1911), *P.gathofi* Molina-Acevedo, 2018, *P.gemmata* (Mohammad, 1973), *P.miroi* Molina-Acevedo, 2018, *P.patriciae* Molina-Acevedo, 2018, *P.purcellana* (Willey, 1904), *P.triantennata* Kim, Soh & Jeong, 2022 and with an undescribed species (*Paucibranchia* sp. 2 of Molina-Acevedo 2018). However, it differs from *P.conferta*, *P.gemmata*, *P.miroi*, *P.patriciae*, *P.purcellana*, and *P.* sp. 2 by the chaetiger on which the branchiae start (chaetiger 14–16 for *P.glemareci* sp. nov. versus chaetiger 22 for *P.gemmata*, chaetiger 10–12 for *P.triantennata* and chaetiger 7 or 8 for the other five species) and from *P.gathofi* by the chaetiger where subacicular hooks start (chaetiger 33–37 for *P.glemareci* sp. nov. versus chaetiger 17–30 for *P.gathofi*).

*Paucibranchiaglemareci* sp. nov. may have been mistaken in the past for *P.bellii* (as *Marphysabellii*), the only name for specimens with pectinate branchiae restricted to the anterior part of the body in the early literature (e.g. Fauvel 1923). Thus, old records of *P.bellii* from offshore area in the Bay of Biscay should be regarded as doubtful.

### ﻿Molecular analyses

**Figure 5.**
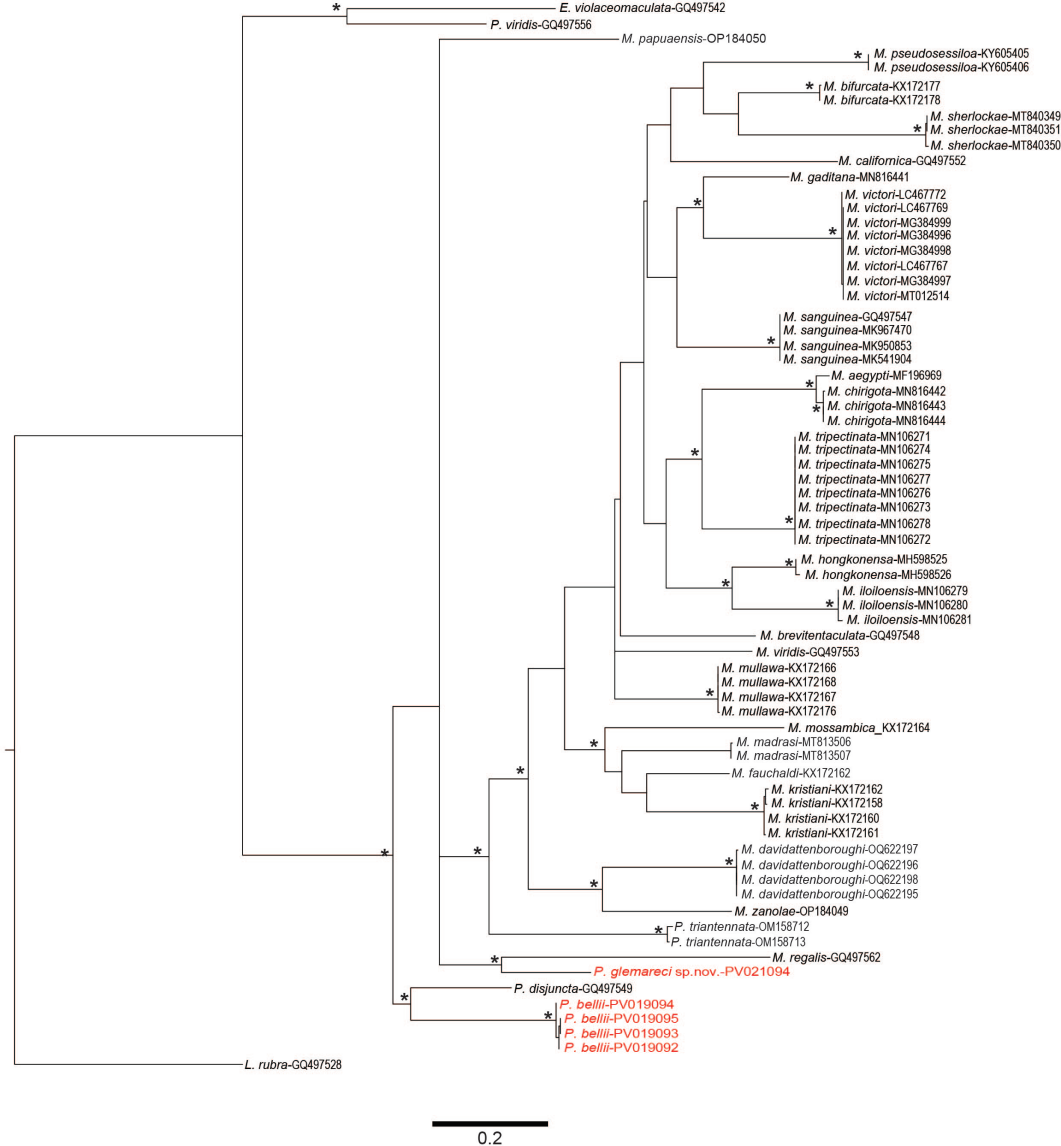
Maximum-likelihood tree of *Marphysa* and *Paucibranchia* species based on COI sequences. Asterisks indicate the bootstrap support values of the ML analysis > 70%. Text in red indicates specimens analysed in this study.

COI gene was successfully sequenced and published at NCBI GenBank for one specimen of the new species *Paucibranchiaglemareci* sp. nov. and for four specimens of *Paucibranchiabellii* collected from the same geographical area (Table 1, Figs 1, 5). The new species *P.glemareci* sp. nov. is clearly different from other species of the *Marphysa*-*Paucibranchia* group for which molecular data are available. With an interspecific distance (K2P) of 19.2%, *P.glemareci* sp. nov. is relatively close to *Marphysaregalis* Verrill, 1900 described from Bermuda. *Paucibranchiaglemareci* sp. nov. lives in the same geographical area as *P.bellii*, and their interspecific distance is 20.6% (standard error: 0.15%) (Fig. 5). *Paucibranchiaglemareci* sp. nov. is separated from the main *Marphysa* clade by a mean intraspecific distance of 22.3% (standard error: 0.2%) (Fig. 5).

Based on our molecular analysis, the status of the genera *Marphysa* and *Paucibranchia* is still not clear. Indeed, *Paucibranchia* species seem to be separated from the main *Marphysa* clade, but *M.regalis* is a sister species of *P.glemareci* sp. nov., and *Marphysapapuaensis* Lavesque, Daffe, Glasby, Hourdez & Hutchings, 2022 is isolated. When they described *M.papuaensis*, Lavesque et al. (2022) hesitated as to which genus it should be assigned to as this species has a limited number of branchial segments. However, the maxillae I (Lavesque et al. 2022: fig. 6D) are more similar to those of *Marphysa* (the base arch lacking a curvature in the basal inner edge) than those of *Paucibranchia* (the base straight with a curvature in the basal inner edge) (Molina-Acevedo 2018). A phylogenetic study based on molecular data of the genera *Marphysa*, *Paucibranchia*, and closely related *Treadwellphysa* Molina-Acevedo & Carrera-Parra, 2017 is really necessary to test their monophyly and placement within Eunicidae (Zanol, pers. comm.).

### ﻿Key to the European species of *Paucibranchia*

**Table:** 

1	Branchiae present over most of the body, prostomium anteriorly bilobed	***Marphysa* Quatrefages, 1865**
–	Branchiae limited to anterior part of the body, prostomium anteriorly rounded ***Paucibranchia* Molina-Acevedo, 2018**	**2**
2	Composed chaetae only spinigers	***P.kinbergi* (McIntosh, 1910)**
–	Composed chaetae only falcigers	**3**
–	Composed chaetae both spinigers and falcigers	**4**
3	Up to four aciculae on pre-branchial chaetigers, presence of reddish spots in preserved specimens	***P.glemareci* sp. nov.**
–	Up to two (three in Mediterranean questionably identified specimens) aciculae on pre-branchial chaetigers. With or without brown colouration	***P.adenensis* (Gravier, 1900)**
4	Branchiae with up to two filaments	***P.fallax* (Marion & Bobretzky, 1875)**
–	Branchiae with six or more filaments	**5**
5	Falcigerous composed chaetae on posterior chaetigers only	***P.cinari* (Kurt-Sahin, 2014)**
–	Falcigerous composed chaetae along body	**6**
6	Spinigerous composed chaetae on anterior body only (up to chaetiger 53 max)	***P.bellii* (Audouin & Milne-Edwards, 1833)**
–	Spinigerous composed chaetae all along body	***P.totospinata* (Lu & Fauchald, 1998)**

## Supplementary Material

XML Treatment for Paucibranchia

XML Treatment for Paucibranchia glemareci

## ﻿Additional information

### Author ORCIDs

Lucas Pinsivy https://orcid.org/0009-0006-6854-1374

Nicolas Lavesque https://orcid.org/0000-0001-5701-2393

Guillemine Daffe https://orcid.org/0000-0002-7085-3151

Flore Daramy https://orcid.org/0000-0002-1141-4554

Pat Hutchings https://orcid.org/0000-0001-7521-3930

### Data availability

All of the data that support the findings of this study are available in the main text or Supplementary Information.

## Supplementary materials

Supplementary material 1 Metadata of specimens examined in the study Data type csv Explanation note Metadata of specimens examined in the study with specimens registration numbers, type status, associated COI sequences in genbank, taxon name, geolocation and depth. https://binary.pensoft.net/file/1284095This dataset is made available under the Open Database License (http://opendatacommons.org/licenses/odbl/1.0/). The Open Database License (ODbL) is a license agreement intended to allow users to freely share, modify, and use this Dataset while maintaining this same freedom for others, provided that the original source and author(s) are credited.Lucas Pinsivy
